# Acquired Hemophilia A Presenting as Intramuscular Hematoma

**DOI:** 10.1177/2324709618817572

**Published:** 2018-12-06

**Authors:** Ghassan Al-Shbool, Anusha Vakiti

**Affiliations:** 1 MedStar Washington Hospital Center, Washington, DC, USA

**Keywords:** acquired hemophilia A, Obizur, cyclophosphamide, rituximab, bleeding disorder

## Abstract

Acquired hemophilia A poses a clinical and diagnostic challenge. Although rare, it is still the most common acquired factor deficiency. We present a case of acquired hemophilia A diagnosed in a 71-year-old female who presented with a right thigh hematoma of acute onset. The diagnosis was established based on the coagulation profile along with factor VIII levels, mixing studies, and inhibitor levels. The patient received multiple lines of therapy including steroids, factor VIIa, Obizur (porcine-derived recombinant factor VIII), followed by multiple cycles of chemotherapy including cyclophosphamide and rituximab.

## Introduction

Acquired hemophilia A (AHA) is considered as a differential diagnosis in any case of acute bleeding in the absence of an underlying clear etiology.^1^ Although a rare hemorrhagic disorder, it is still the most common acquired factor deficiency with incidence about 1 million per year.^2^ It is caused by autoantibodies against factor VIII, known as inhibitors.^3,4^ AHA can be secondary to underlying disease or idiopathic,^5^ and presents in adult women in different clinical scenarios.^6^ Outcome could be fatal mainly during the first few weeks of diagnosis.^7^ Intramuscular bleeding is one of these rare manifestation with few case reports mentioned in the literature.^4^ In this article, we discuss a case of new-onset AHA presenting as right thigh hematoma.

## Case Presentation

**Figure 1.**
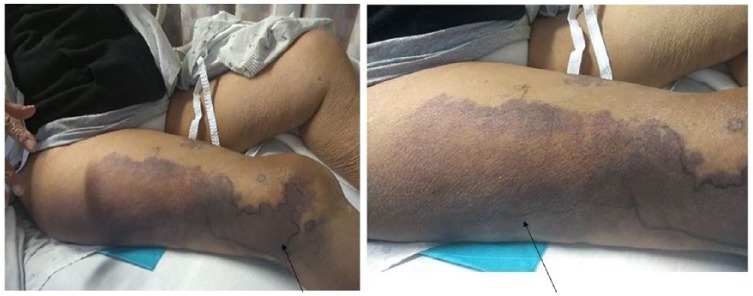
Areas of demarcation showing the right thigh hematoma (arrows).

**Figure 2.**
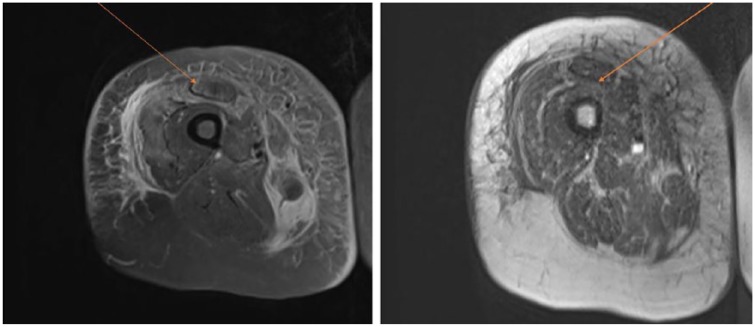
Magnetic resonance imaging showing notable soft tissue edema/inflammation in the right thigh primarily involving the anterior compartment, most advanced in the rectus femoris muscle, to lesser extent the vastus musculature, sartorius, and gracilis. There is suspected hemorrhage within the proximal right rectus femoris muscle but no organized fluid collection/hematoma (arrows).

A 71-year-old female with past medical history of hypertension, abdominal and thoracic aortic aneurysm, and chronic obstructive pulmonary disease presented to the hospital complaining of pain and bruising in right thigh of 6 days duration. She denied any history of abnormal bleeding, family history of bleeding disorders, or use of any anticoagulant medications. Physical examination revealed large subcutaneous hematoma on the anterior aspect of the right thigh, extending posteriorly and laterally (Figure 1). Imaging including ultrasound to rule out deep venous thrombosis was negative, and magnetic resonance imaging showed intramuscular bleeding into the anterior compartment of the right thigh (Figure 2). Laboratory data (Table 1) including complete blood count revealed hemoglobin level of 6.8 g/dL that responded appropriately to 1 unit of blood transfusion. Coagulation profile showed prolonged activated partial thromboplastin time (aPTT) 52.5 seconds, normal prothrombin time, and normal international normalized ratio. Mixing study failed to correct aPTT, factor VIII assay was low at 2%, and inhibitor levels were elevated at 16 Bethesda units (BU) suggestive of AHA. Treatment was started with factor VIIa 5 mg multiple times daily, Obizur (porcine-derived recombinant factor VIII) 15 000 units twice daily, and methylprednisone 80 mg daily without any improvement. Due to the lack of any clinical response to the initial therapy, chemotherapy with cyclophosphamide and rituximab was initiated. The patient received 1 cycle of the combination chemotherapy, followed by second cycle of rituximab monotherapy after 1 week. She was transitioned from methylprednisone to prednisone during the same period. After the second cycle of chemotherapy, factor VIII assay was rechecked and improved to >5%. The thigh hematoma reduced in size, and the patient did not experience any further bleeding during the hospitalization. She was later discharged on a prednisone taper and with an outpatient follow-up with hematology to continue rituximab cycles weekly.

**Table 1.** Laboratory work up during hospitalization.

Laboratory Test	On Admission	On Second Day	On Fifth Day	On Discharge
Hemoglobin (g/dL)	6.8	8.0	9.5	10.5
Platelets	188	186	288	267
Activated thromboplastin time (seconds)	52.5	51.7	42	36
Prothrombin time and international normalized ratio	12.5	13	13.1	12.4
Activated thromboplastin time (seconds) mixing study	51			
Factor VIII assay (%)	2	2	3	11
Factor VIII inhibitor assay (Bethesda units)	16			

## Discussion

AHA is considered an anticoagulation defect secondary to the presence of inhibitory autoantibodies against factor VIII.^1^ The first case of AHA was described in 1904 by Lozner et al, in an elderly male who suffered from major bleeding after a surgery.^8^ It is a rare autoimmune disorder with an annual incidence of 1.5 cases per million per year, considered an underestimation given that most cases are diagnosed later in the course with fatal complications. Its incidence increases with age, and the median age of diagnosis is 75 years.^9^ Many risk factors contribute to AHA, with pregnancy and postpartum being the most common risks (2% to 21%).^9-11^ Other risk factors associated with high incidence of AHA include autoimmune disorders (20%), malignancies (12%), dermatological disorders (2%), and medications. Rarely, it can also be associated with infections and blood transfusions. However, the majority of the cases are still considered idiopathic.^1^

Typically, patients with AHA present with subcutaneous hematomas, mucosal bleeding, intramuscular bleeding, retroperitoneal bleeding, and bleeding post surgery. The hematomas are generally diffuse and very painful. In contrast to congenital hemophilias, joint bleeding is uncommon in AHA.^12,13^ Family history, history of abnormal bleeding, and use of anticoagulation or antiplatelet medications may help differentiate AHA from other bleeding disorders.^14^ In our case, the patient presented with pain in the anterior aspect of the right thigh associated with swelling, erythematic skin changes, and acute normocytic anemia, without any significant family history or risk factors except her age.

The diagnosis of AHA is considered a challenge given its nonspecific presentation and low prevalence across the globe. A general approach to management is to focus on stabilization of the patient initially then evaluate the cause of bleeding starting with platelets counts and coagulation profile. Assessment of coagulation profile shows isolated prolonged aPTT (typically by 2-3 times), which could be due to multiple causes like deficiency of intrinsic coagulation factors (FVIII, IX, XI, XII) and the presence of coagulation factor inhibitors. Therefore, quantitative measurements assays of coagulation factors activity and measurement of coagulation factors inhibitor level are considered to be a crucial diagnostic step.^9,10,12,14^ Finally, mixing study helps differentiate between coagulation factor deficiency and the presence of coagulation factor inhibitor, and it is done by mixing patient’s plasma with normal plasma in 1:1 ratio, which should correct aPTT if the patient had coagulation factor deficiency. However, if the mixing study failed to correct aPTT, this indicates the presence of a coagulation factor deficiency inhibitor. This test should be done after 1 to 2 hours of incubation.^6^ In AHA, coagulation profile demonstrates isolated prolonged aPTT, low factor VIII activity (less than 15% and less than 5% in some registries in Europe^11-15^), elevated coagulation factors inhibitor levels in BU, and mixing study failing to correct aPTT.^6^ Factor VIII activity and inhibitor titers are not correlated to each other and neither to the bleeding severity or treatment outcomes.^1,15-19^ In our case, the patient was admitted with significant anemia with hemoglobin level less than 7 g/dL, coagulation profile demonstrated elevated aPTT, mixing studies failed to correct aPTT, factor VIII activity was found to be low, and inhibitor levels were elevated.

The management of AHA lies in controlling the bleeding initially, preventing further bleeding, treating the underlying cause, and eradicating coagulation factors inhibitors (antibodies).^9,20^ Two main strategies are employed: hemostatic and immunosuppression. The hemostatic arm applies for patients with severe bleeding, includes replacement therapy with recombinant activated factor VIII if the inhibitors level are low (<5 BU), coagulation-bypass agents like recombinant activated factor VII (90 µg/kg every 2-3 hours), and activated prothrombin complex concentrate (50-100 IU/kg every 8-12 hours, maximum of 200 IU/kg/day). For minor bleeding, desmopressin can be used.^19^ The immunosuppression arm uses steroids (prednisone, 1 mg/kg/day for 4-6 weeks), with multiple immunosuppressants (eg, cyclophosphamide cyclosporine, azathioprine, 6-mercaptopurine, vincristine, and mycophenolate mofetil). The main treatment lines that have been used recently includes corticosteroid alone, corticosteroid with cyclophosphamide, and corticosteroids with rituximab,^1,9,21,22^ and should be started immediately without delay to prevent fatal bleeding risk.^9,10,19,20,23^ Remission is achieved when factor VIII activity is more than 50% and factor VIII inhibitor levels become undetected, approximately 4 to 5 weeks after treatment.^1,24^ In our case, the patient received multiple doses of factor VIIa (recombinant activated factor VIII) and steroids for few days without any significant improvement and further was treated with chemotherapy with cyclophosphamide and rituximab. She was eventually discharged on a steroid taper after improvement in factor VIII levels and received weekly rituximab infusions as outpatient.

AHA can be fatal if there is a delay in timely diagnosis, contributing to its high mortality rate.^10^ Prognosis depends on clinical course, severity of bleeding, underlying cause, and the presence of comorbidities. Spontaneous resolution occurs in 25% of the patients, mainly in those with medication or pregnancy-induced AHA.^10,20^ In a recent study by Collins et al, mortality rate of AHA was 41%, with increasing age being the only factor affecting survival, without significant effect of gender, underlying disease, and factors levels.^15^ Relapse is common up to 20% in the first 2 years and thus close follow-up is required.^15,16,25^ One of the approaches suggests measuring factor VIII activity every month for the first 6 months, every 2 to 3 months for the next 6 months, and every 6 months later.^19-21,26^
